# Validity of Intraoral Scans Compared with Plaster Models: An *In-Vivo* Comparison of Dental Measurements and 3D Surface Analysis

**DOI:** 10.1371/journal.pone.0157713

**Published:** 2016-06-15

**Authors:** Fan Zhang, Kyung-Jin Suh, Kyung-Min Lee

**Affiliations:** Department of Orthodontics, School of Dentistry, 4D Dental Research Institute, Chonnam National University, Gwangju, Korea; Monash University, AUSTRALIA

## Abstract

**Purpose:**

Dental measurements have been commonly taken from plaster dental models obtained from alginate impressions can. Through the use of an intraoral scanner, digital impressions now acquire the information directly from the mouth. The purpose of this study was to determine the validity of the intraoral scans compared to plaster models.

**Materials and Methods:**

Two types of dental models (intraoral scan and plaster model) of 20 subjects were included in this study. The subjects had impressions taken of their teeth and made as plaster model. In addition, their mouths were scanned with the intraoral scanner and the scans were converted into digital models. Eight transverse and 16 anteroposterior measurements, 24 tooth heights and widths were recorded on the plaster models with a digital caliper and on the intraoral scan with 3D reverse engineering software. For 3D surface analysis, the two models were superimposed by using best-fit algorithm. The average differences between the two models at all points on the surfaces were computed. Paired *t*-test and Bland-Altman plot were used to determine the validity of measurements from the intraoral scan compared to those from the plaster model.

**Results:**

There were no significant differences between the plaster models and intraoral scans, except for one measurement of lower intermolar width. The Bland-Altman plots of all measurements showed that differences between the two models were within the limits of agreement. The average surface difference between the two models was within 0.10 mm.

**Conclusions:**

The results of the present study indicate that the intraoral scans are clinically acceptable for diagnosis and treatment planning in dentistry and can be used in place of plaster models.

## Introduction

**Fig 1:**
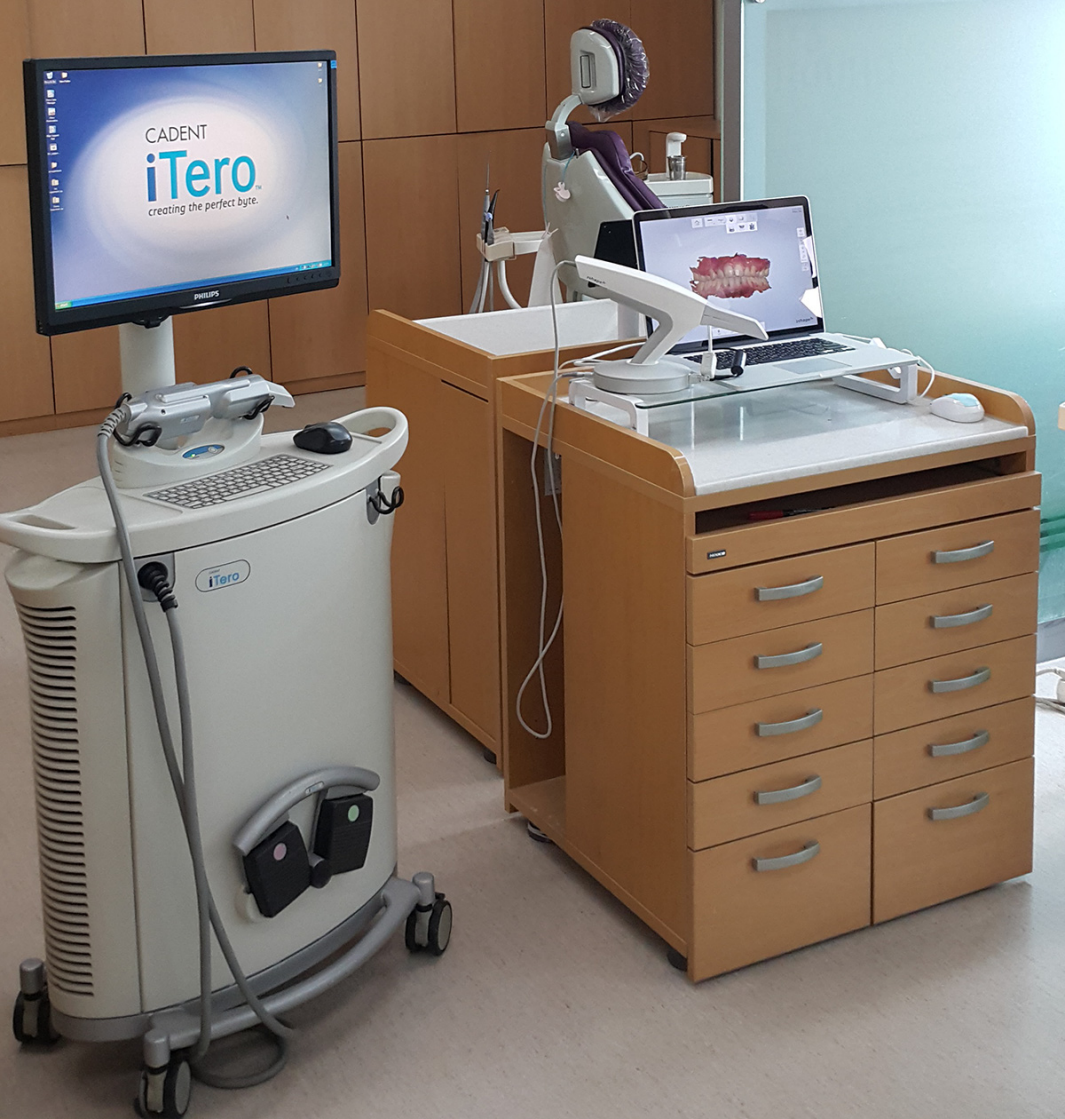
Intraoral scanners used in clinical dental practices.

Since the introduction in dentistry of digital dental impression technique using intraoral scanning devices, there has been a decline in the need for conventional impressions, which requires an impression tray and materials such as alginate. Therefore, clinicians can now acquire the data of patient’s dentition directly by means of intraoral scanner (Fig 1).

In prosthodontics, several authors have investigated the accuracy of intraoral scanners to create prostheses for use in single abutments [1–7] and short-span [8–13]. In their research related to the accuracy of full-arch scans, the authors conducted an in-vitro study using reference model [14–20]. Although single-unit scans demonstrated excellent accuracy, little information is currently available regarding the accuracy of direct (in-vivo) full-arch scanning.

In orthodontics, the full-arch intraoral scan is required for orthodontic diagnosis, treatment planning and evaluation. However, there are a limited number of in-vivo studies on the accuracy of full dentition directly scanned using intraoral scanners. Naidu and Freer [21] compared measurements of tooth widths using intraoral scans versus plaster models. In the other in-vitro study, Grunheid et al. [22] scanned five pairs of plaster models using chair side oral scanner and a model scanner to assess the accuracy of intraoral scan. They scanned the plaster model, not patient’s mouth, to assess the relative accuracy of digital models obtained with the intraoral scanner [22].

Therefore, the purpose of this in-vivo study was to evaluate the accuracy of dental measurements obtained by means of the direct, full-arch intraoral scan as compared with those obtained using the plaster model.

## Materials and Methods

Twenty subjects were included in this study, and the informed consent of each of them was obtained. This study was approved by Chonnam National University Dental Hospital Institutional Review Board (CNUDH-2015-003). All subjects provided their written informed consent to participate in this study. Inclusion criteria were (1) full permanent dentition from the second molar to the contralateral second molar in the jaw; (2) no missing teeth; and (3) no prosthetic restorative teeth. In addition, the subjects with severe crowding and dentofacial deformity were excluded from the study.

Alginate impressions (Cavex Impressional, Cavex Holand BV, Haarlem, The Netherlands) were taken and immediately poured with dental stone (New Plastone II White, GC Corporation Tokyo, Japan). For the digital impression, the subjects’ dentitions were scanned with an intraoral scanner (iTero^®^, Align Technology, San Jose, Calif). All scans with iTero^®^ scanner were recorded by the same examiner (K.M.L) in a predetermined order according to the manufacturer’s recommendation. Scanning started with the upper left second molar continuing to the anterior teeth. Next, the upper right quadrant was scanned, beginning with second molar. Scanning of the mandible started with the right second molar and ended at the central incisor. The lower left quadrant was also scanned starting with the second molar. All scan data were sent to Align Technology, where they were reprocessed and made available for downloading as stereolithography (STL) file on personal computer.

**Fig 2:**
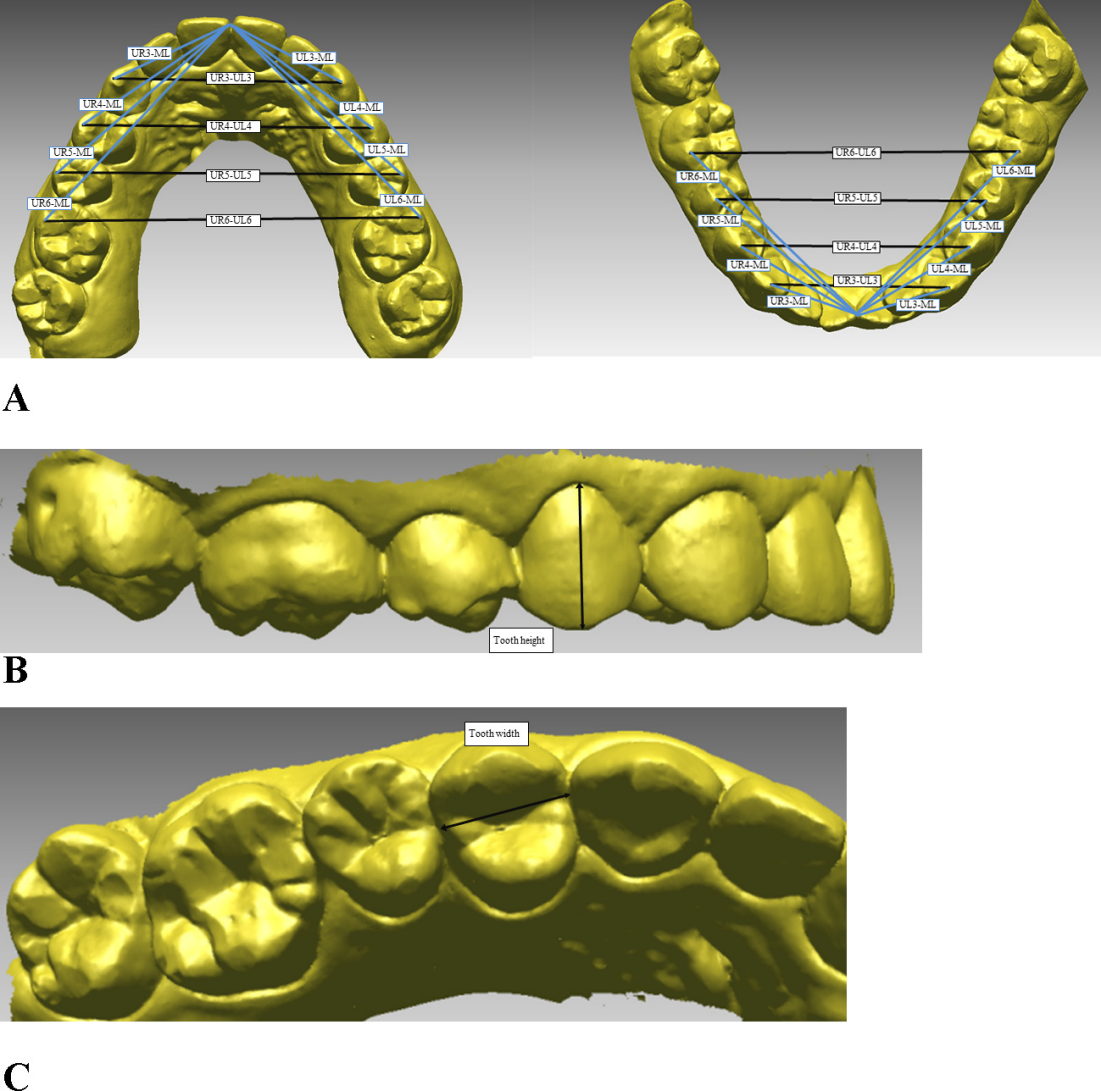
The measurements used in this study. A, Transverse and anteroposterior dimensions of the intra-arch measurements; B, tooth height measurements; C, tooth width measurements.

Intra-arch dimensional dental measurements were recorded using a digital caliper (Mitutoyo, Tokyo, Japan) on the plaster models and using three-dimensional (3D) reverse engineering software (Rapidform^TM^2006, INUS Technology, Seoul, Korea) on the intraoral scans. The intra-arch measurements included the transverse and anteroposterior dimensions of the arch, in addition to tooth heights and widths. Descriptions of the measurements are presented in Fig 2. The tooth heights were measured by selecting the midpoint of the incisal edge (for the incisors), the buccal cusp tip (for the canines and premolars), or the mesiobuccal cusp tip (for the first molars), and the gingival zenith was defined as the most apical point of the marginal gingival scallop of each crown. Tooth widths were measured by selecting the maximum mesiodistal diameter of each crown, which was defined as the distance between the anatomic contact areas when the teeth were correctly aligned (Fig 2). The Federation Dentaire Internationale system was used for tooth numbering.

The plaster models were scanned by using a laser scanner (Orapix, Seoul, Korea). The laser-scanned file of the plaster models was converted to 3D model by using Rapidform^TM^ 2006 program. For 3D surface analysis, the plaster models and intraoral scans were superimposed using the best-fit algorithm in the software. The registration process was performed automatically by the software program using the “register” function. Since the soft tissue areas could increase the error range, the remaining areas were cut out along with the gingival margin to superimpose the crowns alone. The registration process is carried out automatically by the software program using the iterative closest point (ICP) algorithm. The registration uses the surface information from two datasets to calculate the rotation and translation between datasets. The corresponding points and shapes are searched and the distance is minimized after rotation and translation. The error of two surface matching can be evaluated by measuring the 3D Euclidean distances between large numbers of surface points on the two images using the “shell/shell deviation” function of the program. The “shell/shell deviation” displays the color map of the distance deviation between the two shells (point of clouds datasets). In other words, the differences between the two images can be evaluated using color-mapping methods. In the present study, the average differences between intraoral scans and plaster models at all points on the surfaces were computed by means of the “shell/shell deviation” function in the program. The means, standard deviations, minimum, and maximum values of the shell/shell deviation were calculated for the intraoral scan and plaster model.

Paired *t*-test was used to compare the measurements between the plaster models and intraoral scans. Bland-Altman analysis [23] was also used to investigate the agreement between the plaster models and intraoral scans in further detail. The result is a graphical plot that can be used for comparing two models. The graph displays a scatter diagram of the differences plotted against the averages of the two measurements. Horizontal lines are drawn at the mean difference, and at the limits of agreement, which are defined as the mean difference plus and minus 1.96 times the standard deviation of the differences. The mean difference between the plaster models and intraoral scans, the standard deviations of the differences, and the limits of agreement were calculated. To assess the reliability of the measurements, all the measurements were repeated after two weeks, and the mean of the two measurements was used in the statistical analysis. The systematic intraexaminer error between the two measurements was determined using paired *t*-test. Also, the magnitude of the measurement error was assessed by calculating the intraclass correlation coefficient (ICC).

Statistical analyses were performed using version 18.0 of the PASW software package (IBM, Armonk, NY, USA) for paired *t*-test and ICC, and MedCalc (MedCalc Software, Ostend, Belgium) for Bland-Altman analysis.

## Results

The systematic intraexaminer error was found to be statistically insignificant. The ICC measurements indicated excellent reliability with a mean ICC of 0.871 (ICC  = 0.82–0.93).

The means and standard deviations for the intra-arch measurements from the plaster models and intraoral scans, and their differences, are shown in Tables 1, 2, and 3. There was a statistically significant difference in one measurement (LR6-LL6) between the plaster models and intraoral scans. The LR6-LL6 measurement was 45.08 mm for the plaster models and 45.36 mm for the intraoral scans, 0.28 mm larger in the intraoral scans than in the plaster models (Table 1). For the anteroposterior dimension of the arch, there were no significant differences between the plaster models and intraoral scans (Table 1). The differences between the two models were less than 0.2 mm. For the teeth height and width measurements, there were no significant differences between the two models, and the differences in means were less than 0.1 mm (Tables 2 and 3).

**Table 1:** Comparison of the intra-arch measurements (transverse and anteroposterior dimensions) between the plaster models and intraoral scans.

Measurements (mm)	Plaster model		Intraoral scan		Difference		P value
	Mean	SD	Mean	SD	Mean	SD	
**Transverse**							
**UR3-UL3**	35.37	1.71	35.59	1.83	-0.22	0.56	0.174
**UR4-UL4**	42.66	1.8	42.76	1.87	-0.10	0.24	0.159
**UR5-UL5**	49.16	1.92	49.15	1.85	0.01	0.18	0.809
**UR6-UL6**	53.49	2.58	53.57	2.53	-0.08	0.23	0.192
**LR3-LL3**	26.52	2.12	26.59	2.16	-0.07	0.22	0.254
**LR4-LL4**	34.04	1.90	34.26	1.87	-0.22	0.46	0.103
**LR5-LL5**	40.23	2.77	40.31	2.89	-0.08	0.23	0.245
**LR6-LL6**	45.08	2.36	45.36	2.48	-0.28	0.31	0.005*
**Anteroposterior**							
**UR3-ML**	20.01	1.40	19.91	1.25	0.10	0.41	0.393
**UR4-ML**	26.76	1.29	26.83	1.36	-0.07	0.17	0.147
**UR5-ML**	33.07	1.38	33.15	1.42	-0.08	0.17	0.130
**UR6-ML**	38.89	1.41	38.88	1.35	0.01	0.28	0.911
**UL3-ML**	20.31	1.12	20.22	1.14	0.09	0.19	0.086
**UL4-ML**	26.96	1.14	26.89	1.12	0.07	0.21	0.073
**UL5-ML**	33.64	1.38	33.57	1.52	0.07	0.32	0.449
**UL6-ML**	39.17	1.31	39.12	1.29	0.05	0.14	0.231
**LR3-ML**	14.97	1.24	14.90	1.19	0.07	0.26	0.341
**LR4-ML**	20.87	0.97	20.92	1.12	0.05	0.18	0.086
**LR5-ML**	27.26	1.01	27.45	1.10	-0.19	0.73	0.069
**LR6-ML**	32.84	1.18	33.01	1.21	-0.17	0.23	0.066
**LL3-ML**	14.76	1.24	14.74	1.18	0.02	0.20	0.707
**LL4-ML**	20.97	0.85	20.86	0.85	0.11	0.21	0.072
**LL5-ML**	27.43	1.13	27.41	1.27	0.02	0.33	0.790
**LL6-ML**	33.29	1.41	33.23	1.36	0.06	0.22	0.082

*SD*, Standard deviation.

*P* values were obtained from paired t-test.

**Table 2:** Comparison of the tooth height measurements between the plaster models and intraoral scans.

Measurements (mm)	Plaster model		Intraoral scan		Difference		P value
	Mean	SD	Mean	SD	Mean	SD	
**Maxilla**							
** Central incisor, right**	9.84	1.06	9.83	1.04	0.01	0.14	0.752
** Central incisor, left**	9.85	0.97	9.88	0.92	-0.03	0.18	0.523
** Lateral incisor, right**	8.29	0.96	8.28	0.96	0.01	0.19	0.893
** Lateral incisor, left**	8.61	1.00	8.66	0.92	-0.05	0.14	0.162
** Canine, right**	9.85	1.68	9.86	1.62	-0.01	0.20	0.845
** Canine, left**	10.00	1.64	10.02	1.67	-0.02	0.25	0.750
** 1st premolar, right**	8.31	1.00	8.29	1.02	0.02	0.10	0.690
** 1st premolar, left**	7.25	1.00	7.22	1.00	0.02	0.19	0.589
** 2nd premolar, right**	8.41	1.02	8.42	0.98	-0.01	0.19	0.769
** 2nd premolar, left**	7.16	0.87	7.14	0.93	0.02	0.13	0.550
** 1st molar, right**	5.80	0.59	5.77	0.57	0.03	0.16	0.420
** 1st molar, left**	5.85	0.59	5.83	0.64	0.02	0.15	0.705
**Mandible**							
** Central incisor, right**	7.89	0.91	7.91	0.97	-0.02	0.17	0.712
** Central incisor, left**	7.82	0.99	7.80	0.98	0.02	0.22	0.676
** Lateral incisor, right**	8.10	0.77	8.13	0.80	-0.03	0.12	0.367
** Lateral incisor, left**	8.15	0.91	8.18	0.87	-0.03	0.15	0.434
** Canine, right**	9.75	0.93	9.69	0.86	0.06	0.27	0.377
** Canine, left**	9.74	0.89	9.68	0.81	0.06	0.16	0.352
** 1st premolar, right**	8.58	1.01	8.60	0.95	-0.02	0.21	0.723
** 1st premolar, left**	7.22	0.83	7.18	0.83	0.04	0.13	0.290
** 2nd premolar, right**	8.44	1.03	8.40	0.98	0.04	0.23	0.505
** 2nd premolar, left**	7.14	1.00	7.24	0.95	-0.10	0.17	0.056
** 1st molar, right**	6.45	0.66	6.44	0.57	0.01	0.14	0.843
** 1st molar, left**	6.26	0.59	6.25	0.55	0.01	0.14	0.882

*SD*, Standard deviation.

*P* values were obtained from paired t-test.

**Table 3:** Comparison of the tooth width measurements between the plaster models and intraoral scans.

Measurements (mm)	Plaster model		Intraoral scan		Difference		P value
	Mean	SD	Mean	SD	Mean	SD	
**Maxilla**							
** Central incisor, right**	8.20	0.41	8.15	0.34	0.05	0.13	0.207
** Central incisor, left**	8.17	0.54	8.14	0.46	0.03	0.20	0.564
** Lateral incisor, right**	7.15	0.52	6.94	0.46	0.02	0.27	0.134
** Lateral incisor, left**	7.10	0.30	7.04	0.36	0.06	0.21	0.303
** Canine, right**	7.93	0.41	7.84	0.45	0.09	0.20	0.169
** Canine, left**	7.77	0.35	7.76	0.38	0.01	0.20	0.840
** 1st premolar, right**	7.35	0.34	7.35	0.38	0.00	0.16	0.922
** 1st premolar, left**	7.37	0.28	7.35	0.28	0.02	0.11	0.178
** 2nd premolar, right**	7.02	0.36	6.98	0.36	0.04	0.12	0.621
** 2nd premolar, left**	7.05	0.26	7.07	0.23	-0.02	0.15	0.652
** 1st molar, right**	9.98	0.44	9.94	0.50	0.04	0.19	0.425
** 1st molar, left**	9.99	0.50	9.90	0.60	0.09	0.20	0.133
**Mandible**							
** Central incisor, right**	5.36	0.28	5.32	0.29	0.04	0.12	0.236
** Central incisor, left**	5.37	0.34	5.30	0.34	0.07	0.15	0.097
** Lateral incisor, right**	5.96	0.29	5.89	0.26	0.07	0.08	0.099
** Lateral incisor, left**	5.97	0.33	5.99	0.32	-0.02	0.15	0.694
** Canine, right**	6.89	0.48	6.88	0.47	0.01	0.13	0.892
** Canine, left**	6.87.	0.40	6.81	0.39	0.02	0.15	0.634
** 1st premolar, right**	7.21	0.45	7.23	0.38	-0.02	0.19	0.701
** 1st premolar, left**	7.23	0.44	7.15	0.39	0.08	0.17	0.388
** 2nd premolar, right**	7.24	0.35	7.20	0.42	0.04	0.16	0.092
** 2nd premolar, left**	7.16	0.40	7.16	0.42	0.00	0.18	0.920
** 1st molar, right**	11.27	0.62	11.29	0.52	-0.02	0.18	0.605
** 1st molar, left**	11.18	0.53	11.16	0.46	0.02	0.13	0.570

*SD*, Standard deviation.

*P* values were obtained from paired t-test.

The Bland-Altman plots showed that the measurements including transverse and anteroposterior dimensions and the tooth heights and widths between the plaster models and intraoral scans were within the limits of agreement (Table 4).

**Table 4:** Comparison of the measurements between the plaster models and intraoral scans by Bland-Altman analysis.

Measurements	Bias (mm)	Lower limit of agreement (mm)	Upper limit of agreement (mm)
** Transverse**	-0.334	-2.124	1.451
** Anteroposterior**	0.031	2.688	-2.626
** Tooth width**	0.644	-5.231	6.525
** Tooth height**	0.065	-4.191	4.322

As for the 3D analysis, the means, standard deviations, minimum, and maximum values for the average surface differences between intraoral scans and plaster models at all points on the surfaces are shown in Table 5. The average surface differences were within 0.10 mm in the maxilla and mandible. The minimum average surface differences were 0.06 mm in the maxilla and 0.07 mm in the mandible. The maximum average surface differences were 0.13 mm in the maxilla and 0.18 mm in the mandible.

**Table 5:** Shell/shell deviations between the plaster models and intraoral scans.

	Plaster model vs intraoral scan			
	Mean	SD	Minimum	Maximum
**Maxilla (n = 20)**	0.10	0.03	0.06	0.13
**Mandible (n = 20)**	0.09	0.02	0.07	0.18

*SD*, Standard deviation.

## Discussion

Dental measurements have been commonly taken from plaster dental models obtained from alginate impressions. Intraoral access using the conventional alginate impression technique can be a challenge for patients and clinicians with gag reflex, excess saliva, small oral cavities, or large cheeks and tongue. With the conventional technique, the entire impression (tray and material) needs to be in the mouth and has to be stable until the impression material has completely set. Through the use of intraoral scanner, the digital impression technique now acquires the information directly from the mouth. With the digital impression technique, however, the intraoral scanning process can be paused and continued multiple times to ensure that the patient is comfortable during the procedure.

This in-vivo study examined the accuracy of full-arch digital impressions. The intention behind the digital impressions is to replace the conventional impression process and plaster models. For this purpose, Bland-Altman analysis [23] was also used to more thoroughly investigate the agreement between the plaster models and intraoral scans. Based on the Bland-Altman plots, corrected standard deviations were used to account for the two kinds of data. In the present study, as a reference, the plaster model was used as the gold standard. Digital dental models have been used in the orthodontics and restorative dentistry fields for diagnosis, treatment planning, and treatment evaluation [24–26]. Although digital dental models have been used in dentistry since the late 1990s, plaster dental models have still been the gold standard in clinical diagnosis and treatment planning for many years [27–28].

**Fig 3:**
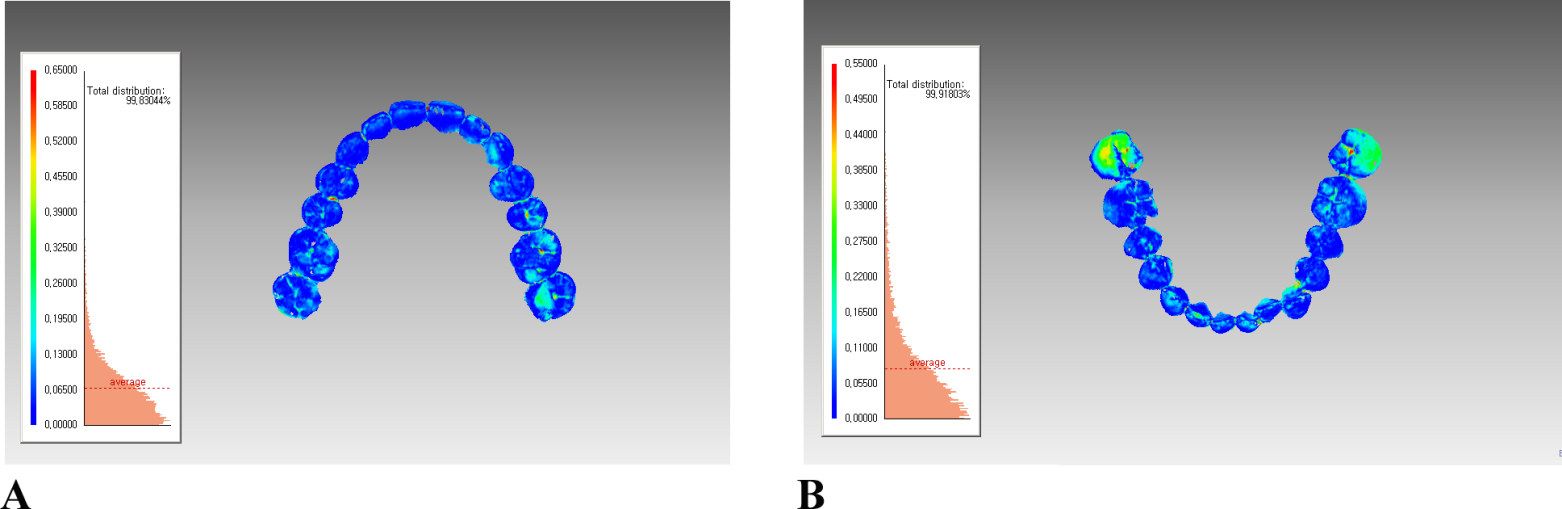
The color-coded visualization charts show the differences between the plaster models and intraoral scans after the registration process. According to the average surface differences shown in the color scale, mandibular molar regions presented discrepancies between the two models.

With regard to the transverse measurements, there was a statistically significant difference in one measurement (LR6-LL6) between the plaster models and intraoral scans. It is not easy for the scanner tip or head to access mandibular posterior molar areas due to the tongue movement and the limit to opening the mouth. This might cause scanning distortion, and consequently, centrifugal expansion. This corresponds with the findings of the 2014 studies, in which the accuracy of the data obtained from intraoral scanners was investigated [18,19]. Patzelt et al. [18] evaluated the accuracy of full-arch stereolithographic and milled casts obtained from the scans of three kinds of intraoral scanners, including iTero. They reported that the highest deviations might occur in the distal areas of the casts [18]. Patzelt et al. [19] evaluated four intraoral scanners, including the iTero, in full-arch scans, as compared with the representative model. Among the four scanners, most of the datasets revealed horizontal expansion in the region of the molars. In our study, the average surface differences shown in color scales, distal area of mandibular presented discrepancies between the two models (Fig 3).

Moreover, intraoral conditions such as saliva, tongue, and limited oral space can also contribute to scanning inaccuracies. Clinicians should keep in mind that scanning distortion might occur in the posterior areas of the mandible. In the clinical setting, careful scanning procedures and additional scans of the mandibular posterior areas might improve scanning accuracy.

## Conclusions

Dental measurements were compared using paired *t*-test and Bland-Altman analysis to determine the validity of the intraoral scan. There were no significant differences between the plaster models and intraoral scans, except for one measurement. The Bland-Altman analysis showed that differences between the two models were within the limits of agreement. The results of the present study indicate that dental measurements from direct intraoral scans are clinically acceptable in clinical dental practices and can be used in place of plaster models.
